# Bis(μ-3-nitro­benzene-1,2-dicarboxyl­ato)-κ^4^ *O* ^1^,*O* ^2^:*O* ^1^,*O* ^1′^;κ^4^ *O* ^1^,*O* ^1′^:*O* ^1^,*O* ^2^-bis­[triaqua­(6-carb­oxy-2-nitro­benzoato-κ^2^ *O* ^1^,*O* ^6^)neodymium(III)] dihydrate

**DOI:** 10.1107/S1600536812042754

**Published:** 2012-10-20

**Authors:** Yin-cheng Chang, Zhi-chao Pei, Qi Shuai

**Affiliations:** a College of Science, Northwest A&F University, Yangling 712100, Shanxi Province, People’s Republic of China

## Abstract

The title complex, [Nd_2_(C_8_H_3_NO_6_)_2_(C_8_H_4_NO_6_)_2_(H_2_O)_6_]·2H_2_O, consists of dimeric units related by an inversion center. The Nd^III^ atom is nine-coordinated by three O atoms from water mol­ecules and six from carboxyl­ate atoms. The 1,2-dicarboxylate acid molecules are in a single and double deprotonation stage and exhibit two coord­in­ation modes, *viz.* μ_2_-(κ^4^, *O* ^1^: *O* ^2^: *O* ^2^: *O* ^3^) and μ_1_-(κ^2^, *O* ^2^: *O* ^3^), which are responsible for the dimeric structure framework. The dimeric structure is then assembled into a three-dimensional supramolecular framework *via* O—H⋯O hydrogen bonds.

## Related literature

For the isotypic La compound, see: Xiong & Qi (2007 ▶).
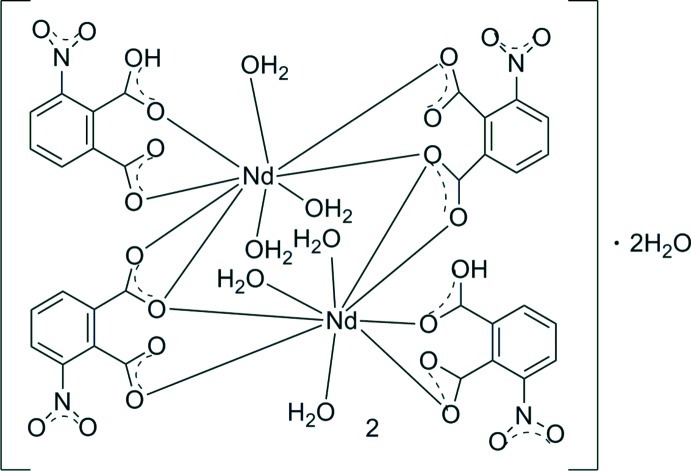


## Experimental

### 

#### Crystal data

[Nd_2__2_(C_8_H_3_NO_6_)_2_(C_8_H_4_NO_6_)(H_2_O)_6_]·2H_2_O
*M* *_r_* = 1271.08
Triclinic, 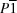

*a* = 8.1460 (7) Å
*b* = 8.8090 (9) Å
*c* = 15.1670 (13) Å
α = 100.434 (1)°
β = 91.106 (1)°
γ = 104.482 (2)°
*V* = 1033.96 (16) Å^3^
*Z* = 1
Mo *K*α radiation
μ = 2.60 mm^−1^
*T* = 298 K
0.16 × 0.14 × 0.10 mm

#### Data collection

Bruker SMART CCD area-detector diffractometer
Absorption correction: multi-scan (*SADABS*; Bruker, 2007 ▶) *T*_min_ = 0.681, *T*_max_ = 0.781
5316 measured reflections
3618 independent reflections
3196 reflections with *I* > 2σ(*I*)
*R*_int_ = 0.030

#### Refinement

*R*[*F* ^2^ > 2σ(*F* ^2^)] = 0.033
*wR*(*F* ^2^) = 0.076
*S* = 1.03
3618 reflections
317 parameters
H-atom parameters constrained
Δρ_max_ = 1.51 e Å^−3^
Δρ_min_ = −0.88 e Å^−3^

### 

Data collection: *SMART* (Bruker, 2007 ▶); cell refinement: *SAINT* (Bruker, 2007 ▶); data reduction: *SAINT*; program(s) used to solve structure: *SHELXS97* (Sheldrick, 2008 ▶); program(s) used to refine structure: *SHELXL97* (Sheldrick, 2008 ▶); molecular graphics: *SHELXTL* (Sheldrick, 2008 ▶); software used to prepare material for publication: *SHELXTL*.

## Supplementary Material

Crystal structure: contains datablock(s) global, I. DOI: 10.1107/S1600536812042754/vn2055sup1.cif

Structure factors: contains datablock(s) I. DOI: 10.1107/S1600536812042754/vn2055Isup2.hkl

Additional supplementary materials:  crystallographic information; 3D view; checkCIF report

## Figures and Tables

**Table 1:** Selected bond lengths (Å)

Nd1—O1	2.489 (3)
Nd1—O3	2.447 (3)
Nd1—O7	2.561 (3)
Nd1—O8	2.564 (3)
Nd1—O8^i^	2.486 (3)
Nd1—O10^i^	2.431 (3)
Nd1—O13	2.554 (3)
Nd1—O14	2.487 (3)
Nd1—O15	2.398 (3)

Symmetry code: (i) 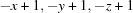.

**Table 2:** Hydrogen-bond geometry (Å, °)

*D*—H⋯*A*	*D*—H	H⋯*A*	*D*⋯*A*	*D*—H⋯*A*
O2—H2⋯O16^ii^	0.82	1.79	2.588 (4)	163
O13—H13*C*⋯O3^i^	0.85	2.19	3.026 (5)	169
O13—H13*D*⋯O10^ii^	0.85	2.12	2.952 (5)	168
O14—H14*B*⋯O7^iii^	0.85	1.98	2.750 (5)	150
O14—H14*C*⋯O4	0.85	2.00	2.782 (4)	152
O15—H15*C*⋯O9^ii^	0.85	1.81	2.654 (4)	177
O15—H15*D*⋯O16^ii^	0.85	2.02	2.867 (5)	177
O16—H16*C*⋯O4	0.85	1.92	2.767 (5)	179
O16—H16*D*⋯O9^iv^	0.85	1.88	2.730 (5)	179

Symmetry codes: (i) 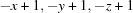; (ii) 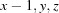; (iii) 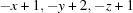; (iv) 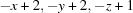.

**Fig. 1.**
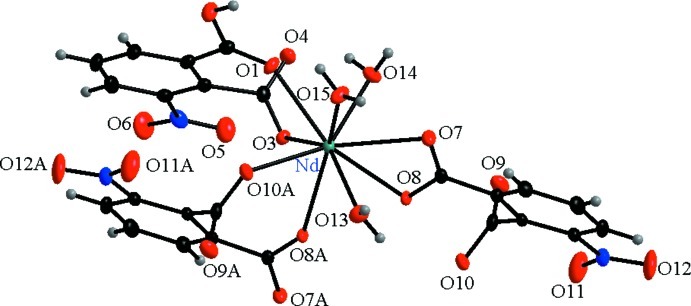
Coordination environment of NdIII atoms; atoms are shown as 30% probability ellipsoids. Symmetry codes: (A) 1 - x, 1 - y, 1 - z;

**Fig. 2.**
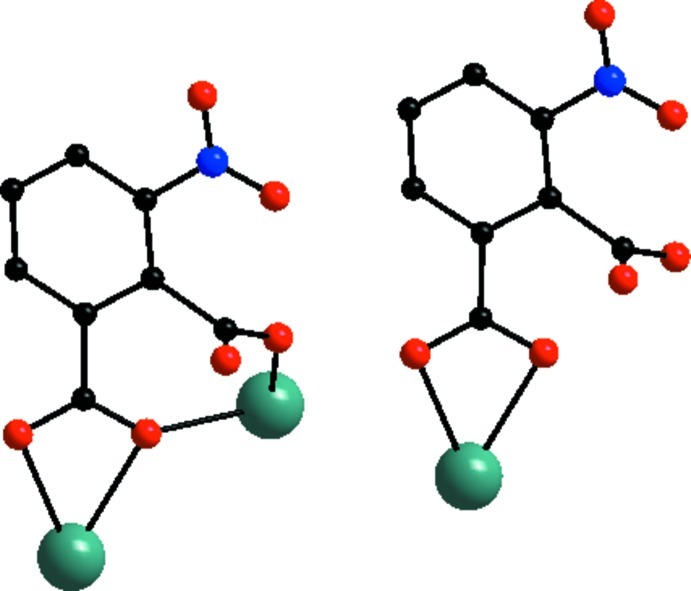
Coordination modes of 3-Nitrobenzene-1,2-dicarboxylic acid ligands in the title complex. Hydrogen atoms are omitted for clarity.

## supplementary crystallographic information

### Comment

We report here a neodymium complex based on 3-nitrobenzene-1,2- dicarboxylic acid ligands. X-ray diffraction crystal structure analysis reveals that the complex forms a structure [Nd_2_(C_8_H_4_NO_6_)_2_(C_8_H_3_NO_6_)_2_ (H_2_O)_6_].2H_2_O, consisting of dimeric units related by an inversion center. The compound is isostructural to the corresponding La compound reported by Xiong & Qi (2007).

In the title complex, the central neodymium center is coordinated by nine oxygen atoms (Fig. 1), the coordination geometry of which can be described as distorted tricapped trigonal-prismatic. Six of the nine coordinating oxygens are from the two coordinating H_2_NPA (3-nitrobenzene-1,2-dicarboxylic acid) ligands and the remaining three from water molecules. 3-Nitrobenzene-1,2-dicarboxylic acid ligands exhibit two coordination modes (Fig. 2), which can be classified as µ_2_-(κ^4^, O^1^: O^2^: O^2^: O^3^) and µ_1_-(κ^2^, O^2^: O^3^). In one unit of the complex, there are four H_2_NPA anions. Two (NPA^2-^) ions are dianionic, so the other (HNPA^-^) anion is monoprotonated to maintain electroneutrality. Correspondingly, two types of coordination modes of H_2_NPA ligands exist in the structure.

### Experimental

A mixture of neodymium chloride hexahydrate (0.03585 g, 0.1 mmol), sodium hydroxide (0.0080 g, 0.2 mmol), 3-nitrobenzene-1,2-dicarboxylic acid (0.0211 g, 0.1 mmol), and CH_3_OH (20 mL) was placed in a Parr Teflon-lined stainless stell vessel (25 ml), which was sealed and heated at 443.15 K for 4 days. Then the vessel was cooled to 373.15 K at a rate of 5 K h^-1^ and subsequently slowly to room temperature. Purple, block single crystals suitable for X-ray diffraction were obtained.

### Refinement

H atoms bonded to C atoms were placed geometrically and treated as riding, with C—H distances 0.93 Å for aryl type H-atoms, respectively with *U*_iso_(H) = 1.2*U*_eq_(C). H atoms attached to O atoms were found in a difference Fourier synthesis and were refined using a riding model, with the O–H distances fixed as initially found and with *U*_iso_(H) values set at 1.2 *U*_eq_(O). Positive and negative residual densities close to oxygen positions are most probably due to different orientational conformations of water molecules or hydroxyl groups. The most likely orientations were retained.

### Crystal data

**Table:**

[Nd_2_(C_8_H_3_NO_6_)_2_(C_8_H_4_NO_6_)_2_(H_2_O)_6_]·2H_2_O	*Z* = 1
*M**_r_* = 1271.08	*F*(000) = 626
Triclinic, *P*1	*D*_x_ = 2.041 Mg m^−^^3^
Hall symbol: -P 1	Mo *K*α radiation, λ = 0.71073 Å
*a* = 8.1460 (7) Å	Cell parameters from 2861 reflections
*b* = 8.8090 (9) Å	θ = 2.6–27.9°
*c* = 15.1670 (13) Å	µ = 2.60 mm^−^^1^
α = 100.434 (1)°	*T* = 298 K
β = 91.106 (1)°	Block, purple
γ = 104.482 (2)°	0.16 × 0.14 × 0.10 mm
*V* = 1033.96 (16) Å^3^	

### Data collection

**Table:**

Bruker SMART CCD area-detector diffractometer	3618 independent reflections
Radiation source: fine-focus sealed tube	3196 reflections with *I* > 2σ(*I*)
Graphite monochromator	*R*_int_ = 0.030
phi and ω scans	θ_max_ = 25.0°, θ_min_ = 2.6°
Absorption correction: multi-scan (*SADABS*; Bruker, 2007)	*h* = −9→8
*T*_min_ = 0.681, *T*_max_ = 0.781	*k* = −10→10
5316 measured reflections	*l* = −14→18

### Refinement

**Table:**

Refinement on *F*^2^	Primary atom site location: structure-invariant direct methods
Least-squares matrix: full	Secondary atom site location: difference Fourier map
*R*[*F*^2^ > 2σ(*F*^2^)] = 0.033	Hydrogen site location: inferred from neighbouring sites
*wR*(*F*^2^) = 0.076	H-atom parameters constrained
*S* = 1.03	*w* = 1/[σ^2^(*F*_o_^2^) + (0.0341*P*)^2^] where *P* = (*F*_o_^2^ + 2*F*_c_^2^)/3
3618 reflections	(Δ/σ)_max_ = 0.001
317 parameters	Δρ_max_ = 1.51 e Å^−^^3^
0 restraints	Δρ_min_ = −0.88 e Å^−^^3^

### Special details

**Table:**

Geometry. All e.s.d.'s (except the e.s.d. in the dihedral angle between two l.s. planes) are estimated using the full covariance matrix. The cell e.s.d.'s are taken into account individually in the estimation of e.s.d.'s in distances, angles and torsion angles; correlations between e.s.d.'s in cell parameters are only used when they are defined by crystal symmetry. An approximate (isotropic) treatment of cell e.s.d.'s is used for estimating e.s.d.'s involving l.s. planes.
Refinement. Refinement of *F*^2^ against ALL reflections. The weighted *R*-factor *wR* and goodness of fit *S* are based on *F*^2^, conventional *R*-factors *R* are based on *F*, with *F* set to zero for negative *F*^2^. The threshold expression of *F*^2^ > σ(*F*^2^) is used only for calculating *R*-factors(gt) *etc*. and is not relevant to the choice of reflections for refinement. *R*-factors based on *F*^2^ are statistically about twice as large as those based on *F*, and *R*- factors based on ALL data will be even larger.

### Fractional atomic coordinates and isotropic or equivalent isotropic displacement parameters (Å^2^)

**Table:**

	*x*	*y*	*z*	*U*_iso_*/*U*_eq_	
Nd1	0.34954 (3)	0.61211 (3)	0.432565 (17)	0.02109 (10)	
N1	0.6299 (5)	0.3586 (5)	0.0945 (3)	0.0357 (11)	
N2	1.0429 (5)	0.8971 (5)	0.8325 (3)	0.0338 (10)	
O1	0.2534 (4)	0.6797 (4)	0.2912 (2)	0.0323 (8)	
O2	0.0666 (4)	0.6809 (4)	0.1849 (2)	0.0387 (9)	
H2	0.0399	0.7439	0.2253	0.058*	
O3	0.5209 (4)	0.5333 (4)	0.3102 (2)	0.0239 (7)	
O4	0.6217 (4)	0.7219 (4)	0.2293 (2)	0.0317 (8)	
O5	0.7245 (5)	0.4265 (5)	0.1614 (3)	0.0525 (11)	
O6	0.6688 (5)	0.2711 (5)	0.0317 (3)	0.0594 (12)	
O7	0.4497 (4)	0.8304 (4)	0.5729 (2)	0.0313 (8)	
O8	0.5879 (4)	0.6447 (3)	0.5528 (2)	0.0222 (7)	
O9	1.0001 (4)	0.8529 (4)	0.6082 (2)	0.0354 (9)	
O10	0.8932 (4)	0.6062 (4)	0.6320 (2)	0.0282 (8)	
O11	1.1186 (5)	0.8219 (6)	0.7825 (3)	0.0686 (15)	
O12	1.1035 (5)	0.9742 (6)	0.9051 (3)	0.0666 (14)	
O13	0.2084 (4)	0.5262 (4)	0.5708 (2)	0.0362 (9)	
H13C	0.2752	0.5118	0.6104	0.043*	
H13D	0.1227	0.5482	0.5960	0.043*	
O14	0.5560 (4)	0.8539 (4)	0.4017 (2)	0.0363 (9)	
H14B	0.5211	0.9371	0.4185	0.044*	
H14C	0.5732	0.8444	0.3461	0.044*	
O15	0.1355 (4)	0.7563 (4)	0.4572 (2)	0.0374 (9)	
H15C	0.0946	0.7860	0.5066	0.045*	
H15D	0.0798	0.7770	0.4150	0.045*	
O16	0.9404 (4)	0.8298 (4)	0.3189 (2)	0.0382 (9)	
H16C	0.8423	0.7975	0.2915	0.046*	
H16D	0.9577	0.9285	0.3414	0.046*	
C1	0.1920 (6)	0.6321 (6)	0.2148 (3)	0.0278 (11)	
C2	0.5315 (6)	0.5899 (5)	0.2390 (3)	0.0249 (11)	
C3	0.2498 (6)	0.5176 (6)	0.1470 (3)	0.0264 (11)	
C4	0.4139 (6)	0.4937 (5)	0.1582 (3)	0.0244 (11)	
C5	0.4606 (6)	0.3880 (5)	0.0893 (3)	0.0292 (11)	
C6	0.3539 (7)	0.3078 (6)	0.0136 (4)	0.0395 (14)	
H6	0.3899	0.2386	−0.0312	0.047*	
C7	0.1944 (7)	0.3323 (6)	0.0059 (4)	0.0397 (14)	
H7	0.1217	0.2795	−0.0444	0.048*	
C8	0.1426 (7)	0.4342 (6)	0.0721 (3)	0.0334 (12)	
H8	0.0335	0.4480	0.0669	0.040*	
C9	0.5563 (6)	0.7666 (5)	0.6009 (3)	0.0225 (10)	
C10	0.9095 (6)	0.7554 (6)	0.6468 (3)	0.0258 (11)	
C11	0.6390 (6)	0.8337 (5)	0.6931 (3)	0.0221 (10)	
C12	0.8033 (6)	0.8239 (5)	0.7179 (3)	0.0208 (10)	
C13	0.8675 (6)	0.8954 (5)	0.8048 (3)	0.0244 (11)	
C14	0.7784 (6)	0.9698 (6)	0.8671 (3)	0.0314 (12)	
H14	0.8264	1.0151	0.9250	0.038*	
C15	0.6166 (7)	0.9762 (6)	0.8425 (4)	0.0354 (13)	
H15	0.5539	1.0254	0.8838	0.042*	
C16	0.5485 (6)	0.9089 (5)	0.7558 (3)	0.0271 (11)	
H16	0.4399	0.9141	0.7391	0.032*	

### Atomic displacement parameters (Å^2^)

**Table:**

	*U*^11^	*U*^22^	*U*^33^	*U*^12^	*U*^13^	*U*^23^
Nd1	0.02379 (15)	0.02005 (14)	0.01938 (16)	0.00804 (10)	−0.00171 (10)	0.00071 (11)
N1	0.037 (3)	0.030 (2)	0.040 (3)	0.009 (2)	0.014 (2)	0.002 (2)
N2	0.033 (3)	0.031 (2)	0.032 (3)	0.005 (2)	−0.009 (2)	−0.002 (2)
O1	0.038 (2)	0.045 (2)	0.0184 (19)	0.0203 (17)	−0.0029 (15)	0.0049 (17)
O2	0.038 (2)	0.046 (2)	0.034 (2)	0.0232 (18)	−0.0095 (17)	−0.0026 (18)
O3	0.0257 (18)	0.0293 (17)	0.0194 (18)	0.0123 (14)	0.0027 (14)	0.0043 (15)
O4	0.0310 (19)	0.0286 (18)	0.032 (2)	0.0014 (15)	0.0011 (15)	0.0071 (16)
O5	0.040 (2)	0.063 (3)	0.053 (3)	0.025 (2)	−0.002 (2)	−0.009 (2)
O6	0.060 (3)	0.069 (3)	0.048 (3)	0.031 (2)	0.017 (2)	−0.013 (2)
O7	0.037 (2)	0.0268 (17)	0.031 (2)	0.0167 (15)	−0.0086 (16)	−0.0019 (16)
O8	0.0302 (18)	0.0204 (16)	0.0161 (17)	0.0115 (14)	−0.0017 (13)	−0.0032 (14)
O9	0.039 (2)	0.0296 (18)	0.037 (2)	0.0069 (16)	0.0173 (17)	0.0048 (17)
O10	0.0263 (18)	0.0225 (17)	0.033 (2)	0.0081 (14)	−0.0031 (15)	−0.0031 (15)
O11	0.045 (3)	0.101 (4)	0.056 (3)	0.039 (3)	−0.016 (2)	−0.020 (3)
O12	0.056 (3)	0.078 (3)	0.052 (3)	0.025 (2)	−0.032 (2)	−0.029 (3)
O13	0.031 (2)	0.050 (2)	0.034 (2)	0.0165 (17)	0.0089 (16)	0.0136 (18)
O14	0.047 (2)	0.0233 (17)	0.039 (2)	0.0116 (16)	0.0102 (17)	0.0047 (16)
O15	0.047 (2)	0.051 (2)	0.024 (2)	0.0328 (19)	0.0043 (16)	0.0055 (18)
O16	0.042 (2)	0.0252 (18)	0.046 (2)	0.0133 (16)	−0.0067 (18)	−0.0017 (17)
C1	0.027 (3)	0.029 (3)	0.028 (3)	0.008 (2)	0.002 (2)	0.009 (2)
C2	0.024 (3)	0.028 (3)	0.023 (3)	0.013 (2)	0.003 (2)	−0.003 (2)
C3	0.028 (3)	0.030 (3)	0.023 (3)	0.007 (2)	0.000 (2)	0.009 (2)
C4	0.027 (3)	0.024 (2)	0.022 (3)	0.005 (2)	0.003 (2)	0.006 (2)
C5	0.039 (3)	0.024 (2)	0.026 (3)	0.008 (2)	0.005 (2)	0.009 (2)
C6	0.061 (4)	0.030 (3)	0.024 (3)	0.008 (3)	0.003 (3)	−0.001 (2)
C7	0.049 (4)	0.037 (3)	0.027 (3)	0.006 (3)	−0.015 (3)	0.000 (3)
C8	0.041 (3)	0.030 (3)	0.029 (3)	0.011 (2)	−0.011 (2)	0.003 (2)
C9	0.022 (2)	0.022 (2)	0.021 (3)	0.002 (2)	0.001 (2)	0.002 (2)
C10	0.022 (3)	0.031 (3)	0.021 (3)	0.010 (2)	−0.005 (2)	−0.005 (2)
C11	0.026 (3)	0.020 (2)	0.020 (3)	0.0053 (19)	0.002 (2)	0.002 (2)
C12	0.024 (2)	0.017 (2)	0.019 (3)	0.0029 (19)	−0.0009 (19)	0.001 (2)
C13	0.030 (3)	0.019 (2)	0.023 (3)	0.006 (2)	−0.001 (2)	0.001 (2)
C14	0.041 (3)	0.030 (3)	0.017 (3)	0.004 (2)	0.001 (2)	−0.002 (2)
C15	0.047 (3)	0.029 (3)	0.028 (3)	0.013 (2)	0.010 (2)	−0.004 (2)
C16	0.026 (3)	0.026 (2)	0.029 (3)	0.011 (2)	0.004 (2)	0.000 (2)

### Geometric parameters (Å, º)

**Table:**

Nd1—O1	2.489 (3)	O14—H14B	0.8500
Nd1—O3	2.447 (3)	O14—H14C	0.8500
Nd1—O7	2.561 (3)	O15—H15C	0.8500
Nd1—O8	2.564 (3)	O15—H15D	0.8500
Nd1—O8^i^	2.486 (3)	O16—H16C	0.8500
Nd1—O10^i^	2.431 (3)	O16—H16D	0.8499
Nd1—O13	2.554 (3)	C1—C3	1.475 (7)
Nd1—O14	2.487 (3)	C2—C4	1.520 (6)
Nd1—O15	2.398 (3)	C3—C8	1.390 (6)
N1—O6	1.212 (5)	C3—C4	1.416 (6)
N1—O5	1.225 (6)	C4—C5	1.394 (7)
N1—C5	1.469 (6)	C5—C6	1.391 (7)
N2—O11	1.197 (6)	C6—C7	1.376 (8)
N2—O12	1.208 (5)	C6—H6	0.9300
N2—C13	1.477 (6)	C7—C8	1.368 (7)
O1—C1	1.211 (6)	C7—H7	0.9300
O2—C1	1.306 (6)	C8—H8	0.9300
O2—H2	0.8200	C9—C11	1.491 (6)
O3—C2	1.265 (6)	C10—C12	1.528 (6)
O4—C2	1.246 (5)	C11—C16	1.389 (6)
O7—C9	1.252 (5)	C11—C12	1.411 (6)
O8—C9	1.270 (5)	C12—C13	1.384 (6)
O8—Nd1^i^	2.486 (3)	C13—C14	1.375 (7)
O9—C10	1.228 (6)	C14—C15	1.381 (7)
O10—C10	1.265 (5)	C14—H14	0.9300
O10—Nd1^i^	2.431 (3)	C15—C16	1.383 (7)
O13—H13C	0.8500	C15—H15	0.9300
O13—H13D	0.8500	C16—H16	0.9300
			
O15—Nd1—O10^i^	82.46 (11)	Nd1—O14—H14C	111.0
O15—Nd1—O3	137.86 (11)	H14B—O14—H14C	109.2
O10^i^—Nd1—O3	90.90 (11)	Nd1—O15—H15C	127.9
O15—Nd1—O8^i^	142.35 (11)	Nd1—O15—H15D	123.5
O10^i^—Nd1—O8^i^	71.22 (10)	H15C—O15—H15D	108.3
O3—Nd1—O8^i^	70.52 (10)	H16C—O16—H16D	108.4
O15—Nd1—O14	90.69 (12)	O1—C1—O2	121.7 (5)
O10^i^—Nd1—O14	143.71 (12)	O1—C1—C3	124.5 (4)
O3—Nd1—O14	70.66 (11)	O2—C1—C3	113.8 (4)
O8^i^—Nd1—O14	126.35 (11)	O4—C2—O3	126.7 (4)
O15—Nd1—O1	68.22 (11)	O4—C2—C4	115.8 (4)
O10^i^—Nd1—O1	73.83 (11)	O3—C2—C4	117.3 (4)
O3—Nd1—O1	69.96 (10)	C8—C3—C4	120.4 (5)
O8^i^—Nd1—O1	125.78 (10)	C8—C3—C1	119.6 (4)
O14—Nd1—O1	70.56 (12)	C4—C3—C1	120.1 (4)
O15—Nd1—O13	75.73 (11)	C5—C4—C3	116.2 (4)
O10^i^—Nd1—O13	76.95 (11)	C5—C4—C2	124.1 (4)
O3—Nd1—O13	143.02 (11)	C3—C4—C2	119.6 (4)
O8^i^—Nd1—O13	72.49 (10)	C6—C5—C4	123.0 (5)
O14—Nd1—O13	135.72 (11)	C6—C5—N1	117.1 (5)
O1—Nd1—O13	135.80 (11)	C4—C5—N1	119.9 (4)
O15—Nd1—O7	72.57 (11)	C7—C6—C5	119.0 (5)
O10^i^—Nd1—O7	141.54 (11)	C7—C6—H6	120.5
O3—Nd1—O7	127.18 (11)	C5—C6—H6	120.5
O8^i^—Nd1—O7	112.88 (10)	C8—C7—C6	120.1 (5)
O14—Nd1—O7	66.79 (11)	C8—C7—H7	120.0
O1—Nd1—O7	120.33 (11)	C6—C7—H7	120.0
O13—Nd1—O7	68.93 (11)	C7—C8—C3	121.3 (5)
O15—Nd1—O8	121.59 (10)	C7—C8—H8	119.4
O10^i^—Nd1—O8	133.76 (10)	C3—C8—H8	119.4
O3—Nd1—O8	92.65 (10)	O7—C9—O8	120.4 (4)
O8^i^—Nd1—O8	66.76 (11)	O7—C9—C11	118.2 (4)
O14—Nd1—O8	79.62 (11)	O8—C9—C11	121.4 (4)
O1—Nd1—O8	149.00 (11)	O9—C10—O10	126.6 (4)
O13—Nd1—O8	72.89 (11)	O9—C10—C12	115.4 (4)
O7—Nd1—O8	50.55 (9)	O10—C10—C12	117.9 (4)
O6—N1—O5	124.0 (5)	C16—C11—C12	119.8 (4)
O6—N1—C5	118.1 (5)	C16—C11—C9	117.6 (4)
O5—N1—C5	117.9 (4)	C12—C11—C9	122.6 (4)
O11—N2—O12	122.9 (5)	C13—C12—C11	116.8 (4)
O11—N2—C13	118.9 (4)	C13—C12—C10	122.9 (4)
O12—N2—C13	118.2 (4)	C11—C12—C10	119.8 (4)
C1—O1—Nd1	147.6 (3)	C14—C13—C12	123.6 (4)
C1—O2—H2	109.5	C14—C13—N2	117.4 (4)
C2—O3—Nd1	122.9 (3)	C12—C13—N2	119.0 (4)
C9—O7—Nd1	94.6 (3)	C13—C14—C15	119.0 (5)
C9—O8—Nd1^i^	140.4 (3)	C13—C14—H14	120.5
C9—O8—Nd1	94.0 (3)	C15—C14—H14	120.5
Nd1^i^—O8—Nd1	113.24 (11)	C14—C15—C16	119.4 (5)
C10—O10—Nd1^i^	129.9 (3)	C14—C15—H15	120.3
Nd1—O13—H13C	115.0	C16—C15—H15	120.3
Nd1—O13—H13D	130.1	C15—C16—C11	121.4 (5)
H13C—O13—H13D	108.7	C15—C16—H16	119.3
Nd1—O14—H14B	111.1	C11—C16—H16	119.3
			
O15—Nd1—O1—C1	−122.1 (6)	O4—C2—C4—C5	88.0 (6)
O10^i^—Nd1—O1—C1	−33.8 (6)	O3—C2—C4—C5	−96.1 (6)
O3—Nd1—O1—C1	63.2 (6)	O4—C2—C4—C3	−87.7 (5)
O8^i^—Nd1—O1—C1	17.7 (6)	O3—C2—C4—C3	88.2 (5)
O14—Nd1—O1—C1	139.0 (6)	C3—C4—C5—C6	−0.6 (7)
O13—Nd1—O1—C1	−84.4 (6)	C2—C4—C5—C6	−176.4 (5)
O7—Nd1—O1—C1	−174.6 (6)	C3—C4—C5—N1	178.8 (4)
O8—Nd1—O1—C1	122.5 (6)	C2—C4—C5—N1	3.0 (7)
O15—Nd1—O3—C2	12.2 (4)	O6—N1—C5—C6	2.4 (7)
O10^i^—Nd1—O3—C2	91.9 (3)	O5—N1—C5—C6	−179.0 (5)
O8^i^—Nd1—O3—C2	161.6 (3)	O6—N1—C5—C4	−177.0 (5)
O14—Nd1—O3—C2	−56.2 (3)	O5—N1—C5—C4	1.6 (7)
O1—Nd1—O3—C2	19.5 (3)	C4—C5—C6—C7	−0.4 (8)
O13—Nd1—O3—C2	161.1 (3)	N1—C5—C6—C7	−179.8 (5)
O7—Nd1—O3—C2	−94.0 (3)	C5—C6—C7—C8	0.0 (8)
O8—Nd1—O3—C2	−134.2 (3)	C6—C7—C8—C3	1.5 (8)
O15—Nd1—O7—C9	161.9 (3)	C4—C3—C8—C7	−2.5 (8)
O10^i^—Nd1—O7—C9	109.9 (3)	C1—C3—C8—C7	177.1 (5)
O3—Nd1—O7—C9	−60.6 (3)	Nd1—O7—C9—O8	7.1 (4)
O8^i^—Nd1—O7—C9	21.7 (3)	Nd1—O7—C9—C11	−170.4 (3)
O14—Nd1—O7—C9	−99.6 (3)	Nd1^i^—O8—C9—O7	−141.9 (4)
O1—Nd1—O7—C9	−147.5 (3)	Nd1—O8—C9—O7	−7.1 (4)
O13—Nd1—O7—C9	80.8 (3)	Nd1^i^—O8—C9—C11	35.6 (7)
O8—Nd1—O7—C9	−3.9 (2)	Nd1—O8—C9—C11	170.4 (4)
O15—Nd1—O8—C9	−12.1 (3)	Nd1^i^—O10—C10—O9	−111.5 (5)
O10^i^—Nd1—O8—C9	−124.2 (3)	Nd1^i^—O10—C10—C12	66.6 (5)
O3—Nd1—O8—C9	142.1 (3)	O7—C9—C11—C16	27.8 (6)
O8^i^—Nd1—O8—C9	−150.5 (3)	O8—C9—C11—C16	−149.7 (4)
O14—Nd1—O8—C9	72.3 (3)	O7—C9—C11—C12	−151.8 (4)
O1—Nd1—O8—C9	88.1 (3)	O8—C9—C11—C12	30.7 (7)
O13—Nd1—O8—C9	−72.6 (3)	C16—C11—C12—C13	−1.6 (6)
O7—Nd1—O8—C9	3.9 (2)	C9—C11—C12—C13	178.0 (4)
O15—Nd1—O8—Nd1^i^	138.38 (13)	C16—C11—C12—C10	−174.2 (4)
O10^i^—Nd1—O8—Nd1^i^	26.3 (2)	C9—C11—C12—C10	5.4 (7)
O3—Nd1—O8—Nd1^i^	−67.47 (13)	O9—C10—C12—C13	−82.4 (6)
O8^i^—Nd1—O8—Nd1^i^	0.0	O10—C10—C12—C13	99.3 (5)
O14—Nd1—O8—Nd1^i^	−137.26 (14)	O9—C10—C12—C11	89.8 (5)
O1—Nd1—O8—Nd1^i^	−121.39 (18)	O10—C10—C12—C11	−88.6 (5)
O13—Nd1—O8—Nd1^i^	77.86 (13)	C11—C12—C13—C14	1.8 (7)
O7—Nd1—O8—Nd1^i^	154.34 (19)	C10—C12—C13—C14	174.1 (5)
Nd1—O1—C1—O2	131.8 (5)	C11—C12—C13—N2	−176.4 (4)
Nd1—O1—C1—C3	−48.8 (9)	C10—C12—C13—N2	−4.0 (7)
Nd1—O3—C2—O4	79.5 (5)	O11—N2—C13—C14	173.7 (5)
Nd1—O3—C2—C4	−95.9 (4)	O12—N2—C13—C14	−6.0 (7)
O1—C1—C3—C8	160.5 (5)	O11—N2—C13—C12	−8.0 (7)
O2—C1—C3—C8	−20.1 (7)	O12—N2—C13—C12	172.3 (5)
O1—C1—C3—C4	−19.9 (7)	C12—C13—C14—C15	−0.8 (8)
O2—C1—C3—C4	159.5 (4)	N2—C13—C14—C15	177.4 (4)
C8—C3—C4—C5	2.0 (7)	C13—C14—C15—C16	−0.4 (8)
C1—C3—C4—C5	−177.6 (4)	C14—C15—C16—C11	0.5 (8)
C8—C3—C4—C2	178.0 (4)	C12—C11—C16—C15	0.5 (7)
C1—C3—C4—C2	−1.6 (7)	C9—C11—C16—C15	−179.1 (4)

Symmetry code: (i) −*x*+1, −*y*+1, −*z*+1.

### Hydrogen-bond geometry (Å, º)

**Table:**

*D*—H···*A*	*D*—H	H···*A*	*D*···*A*	*D*—H···*A*
O2—H2···O16^ii^	0.82	1.79	2.588 (4)	163
O13—H13*C*···O3^i^	0.85	2.19	3.026 (5)	169
O13—H13*D*···O10^ii^	0.85	2.12	2.952 (5)	168
O14—H14*B*···O7^iii^	0.85	1.98	2.750 (5)	150
O14—H14*C*···O4	0.85	2.00	2.782 (4)	152
O15—H15*C*···O9^ii^	0.85	1.81	2.654 (4)	177
O15—H15*D*···O16^ii^	0.85	2.02	2.867 (5)	177
O16—H16*C*···O4	0.85	1.92	2.767 (5)	179
O16—H16*D*···O9^iv^	0.85	1.88	2.730 (5)	179

Symmetry codes: (i) −*x*+1, −*y*+1, −*z*+1; (ii) *x*−1, *y*, *z*; (iii) −*x*+1, −*y*+2, −*z*+1; (iv) −*x*+2, −*y*+2, −*z*+1.
